# Time Moderates the interplay between 5-HTTLPR and stress on depression risk: gene x environment interaction as dynamic process

**DOI:** 10.1192/j.eurpsy.2023.515

**Published:** 2023-07-19

**Authors:** C. Delli Colli, S. Poggini, M. Borgi, B. Vai, F. Cirulli, B. W. J. H. Penninx, F. Benedetti, F. Chiarotti, I. Branchi

**Affiliations:** ^1^Center for Behavioral Sciences and Mental Health, Italian Institute of Health, Rome; ^2^Psychiatry and Clinical Psychobiology, Division of Neuroscience, IRCCS Ospedale San Raffaele, Milan, Italy; ^3^Department of Psychiatry, Amsterdam UMC, Vrije Universiteit Amsterdam, Amsterdam, Netherlands

## Abstract

**Introduction:**

The role of the interaction between the serotonin-transporter-linked promoter region (5HTTLPR) and stressful condition in determining the vulnerability to depression has been widely investigated. Nevertheless, empirical research provides contrasting findings. Recently, the differential susceptibility to environment model proposed a conceptual shift respect to the classical interpretation of 5-HTTLPR: viewing the short (s) and the long (l) allele not as associated to different traits of vulnerability (respectively vulnerable or not), but determining different plasticity levels (respectively, more and less plasticity) and, thus, different susceptibilities to the environment (respectively, high and low susceptibility).

**Objectives:**

As 5-HTTLPR is involved in plasticity, the main goal of the present study is to demonstrate that the interaction between the polymorphism and stress emerges when assessing its effects according to temporal factors in a **dynamic process perspective**.

**Methods:**

We explored our hypothesis, exploiting a meta analytic approach. We searched PubMed, PsychoINFO, Scopus and EMBASE databases and 1096 studies were identified and screened, resulting in 22 studies to be included in the meta-analyses. We applied the DerSimonian and Laird random-effects model to estimate crude odds ratios for risk of depression according to 5HTTLPR and we assessed heterogeneity using the I² and Cochran’s Q statistic. We stratified the staties according to (i) stress duration (i.e., chronic vs. acute stress) and (ii) time elapsed between the end of the stressful condition and the assessment of depression (i.e., within one year vs. more than one year).

**Results:**

When stratifying for the duration of stress, the effect of the 5-HTTLPR x stress interaction emerged only in the case of chronic stress (OR 1.43, 95%IC 1.16-1.77, I²= 52%, Q=25.25; Figure 1), with a significant subgroup difference (p=0.004). The stratification according to time interval revealed a significant interaction only for intervals within one year (OR 1.23, 95%IC 1.03-1-46, I²= 67%, Q=39.35), though no difference between subgroups was found. The critical role of time interval clearly emerged when considering only chronic stress: a significant effect of the 5-HTTLPR and stress interaction was confirmed exclusively within one year (OR 1.53, 95%IC 1.17-2.02, I²= 45%, Q=10.94; Figure 2) and a significant subgroup difference was found (p=0.01).

**Image:**

**Figure:**
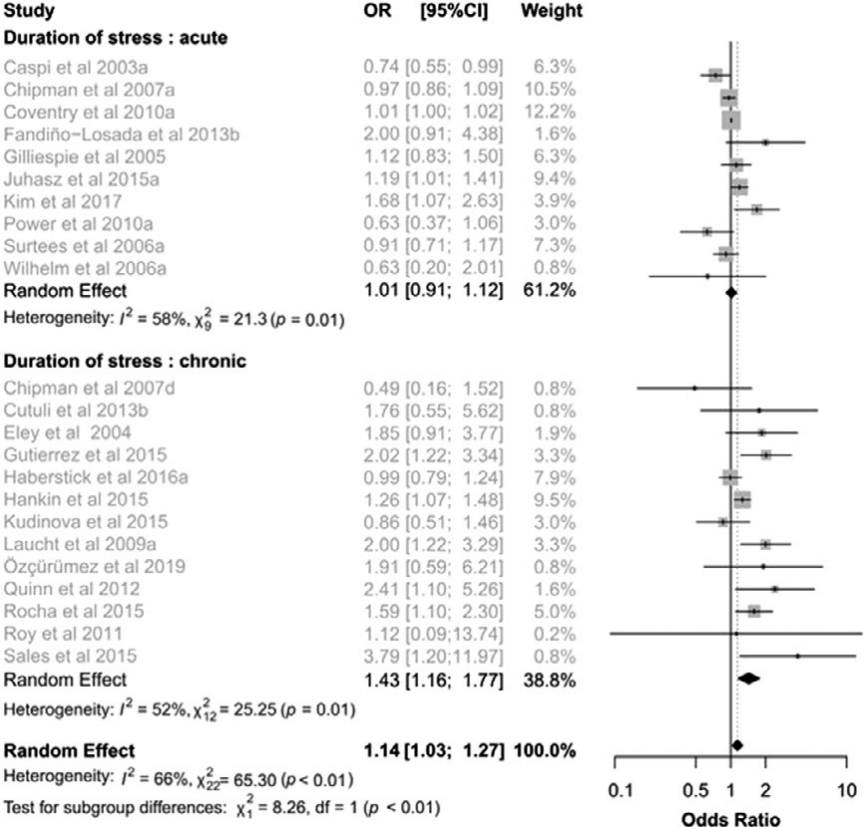

**Image 2:**

**Figure:**
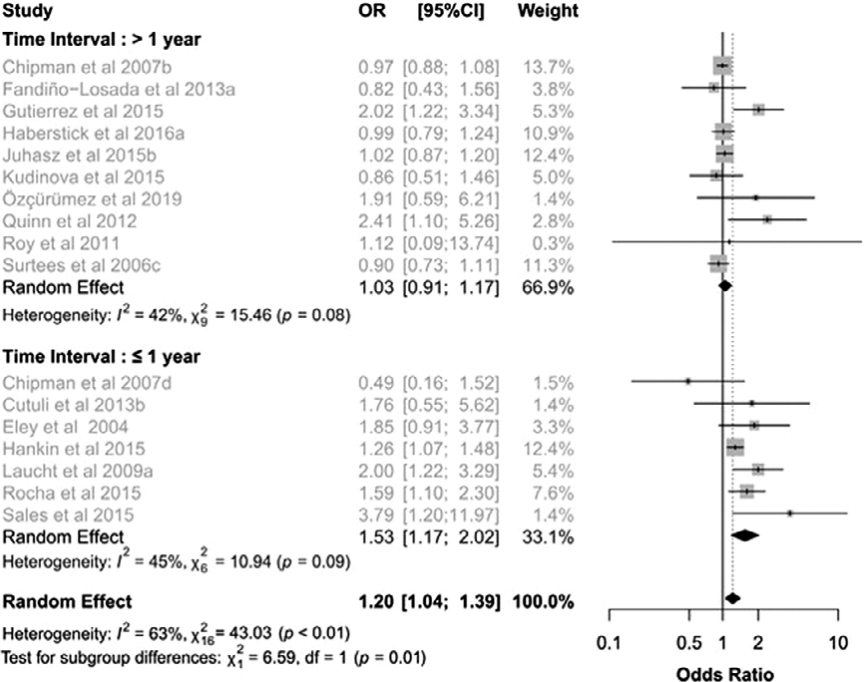

**Conclusions:**

Our results show that the 5-HTTLPR x stress interaction is a dynamic process, producing different effects at different time-points, and indirectly confirm that s-allele carriers are both at higher risk and more capable to recover from depression. Overall, these findings expand the current view of the interplay between 5-HTTLPR and stress adding the temporal dimension, resulting in a three-way interaction: **gene x environment x time**.

**Disclosure of Interest:**

None Declared

